# Time Course Analysis of Motor Excitability in a Response Inhibition Task According to the Level of Hyperactivity and Impulsivity in Children with ADHD

**DOI:** 10.1371/journal.pone.0046066

**Published:** 2012-09-25

**Authors:** Thomas Hoegl, Hartmut Heinrich, Wolfgang Barth, Friedrich Lösel, Gunther H. Moll, Oliver Kratz

**Affiliations:** 1 Department of Child and Adolescent Mental Health, University of Erlangen-Nürnberg, Erlangen, Germany; 2 Heckscher-Klinikum München, München, Germany; 3 Institute of Psychology, University of Erlangen-Nürnberg, Erlangen, Germany; University of Bologna, Italy

## Abstract

Short interval intracortical inhibition (SICI) of motor cortex, measured by transcranial magnetic stimulation (TMS) in a passive (resting) condition, has been suggested as a neurophysiological marker of hyperactivity in attention-deficit/hyperactivity disorder (ADHD). The aim of this study was to determine motor excitability in a go/nogo task at stages of response preparation, activation and suppression in children with ADHD, depending on the level of hyperactivity and impulsivity. Motor evoked potentials were recorded in 29 typically developing children and 43 children with ADHD (subdivided in two groups with higher and lower levels of hyperactivity/impulsivity; H/I-high and H/I-low). In the H/I-high group, SICI was markedly reduced in the resting condition and during response preparation. Though these children were able to increase SICI when inhibiting a response, SICI was still reduced compared to typically developing children. Interestingly, SICI at rest and during response activation were comparable, which may be associated with their hypermotoric behaviour. In the H/I-low group, response activation was accompanied by a pronounced decrease of SICI, indicating reduced motor control in the context of a fast motor response. In summary, different excitability patterns were obtained for the three groups allowing a better understanding of dysfunctional response activation and inhibition processes within the motor cortex in children with ADHD.

## Introduction

Attention-deficit/hyperactivity disorder (ADHD) is one of the most frequently diagnosed child psychiatric disorders with a prevalence rate of about 5% in all school-aged children [1]. It is marked by age-inappropriate levels of inattention, impulsivity and hyperactivity. The most striking symptom is hyperactivity that affects the children and their environments in a mostly negative way.

Barkley [2] suggested that dysfunctional inhibitory processes lead to deficits in a set of other executive functions that accumulate into the behaviour associated with ADHD. Response inhibition refers to the interruption and suppression of inappropriate responses in order to adjust flexibly to changing claims and settings [3], [4] and was thought to be the core dysfunction in ADHD and the key to a better understanding and interpretation of this disorder. In accordance with this view, meta-analyses indicate that performance is impaired in ADHD individuals concerning response inhibition [5], [6]. However, moderate effect sizes along with studies that found no differences compared to healthy controls [7] suggest that weaknesses in executive functions are neither necessary nor sufficient to explain all cases of ADHD [8]. These inconsistent results are not surprising considering other factors, such as the heterogeneity of the disorder and differences in task difficulties [5], [6], [9]. Furthermore, there is not a one-to-one correspondence between genes and certain aspects of ADHD; therefore, specific executive dysfunctions are not necessarily involved.

The misleading concept of a common core to ADHD has been superseded by models of pathophysiological heterogeneity that propose multi-causal pathways to this disorder with each mediated by a different combination of neurophysiological deficits. Dysfunction in response inhibition underpins only one of several pathways to ADHD [10]. Consistent with this proposal, Nigg et al. [11] estimated that only 35 to 50% of children with ADHD Combined type have deficits in response inhibition. This executive deficit is potentially one of several endophenotypes that could predispose a person towards developing ADHD [12], [13].

Beside the inhibitory pathway (caused by mesocortical deficits), the dual-pathway model proposes a motivational trail affected by mesolimbic dysfunctions. Solanto [14] identified a lack of correlation between measures of response inhibition and delay aversion in children with ADHD. This finding implies that these two paradigms tap different components of the disorder, which indicates the existence of independent endophenotypes. Accordingly, averaging at the level of clinical symptoms will not meet the requirements of ADHD as a heterogeneous disorder [10].

At the performance level, successful inhibition might mask underlying brain dysfunctions, as affected children either increase effort or develop compensatory strategies: a shift from behaviour to brain processes is required [10].

One neurophysiological theory proposes that motor hyperactivity might mainly result from insufficient motor facilitation, insufficient motor inhibition or a dysfunctional interaction between both phenomena within cortico-striatal-thalamo-cortical motor circuits in the context of deficits in behavioural inhibition [15]. Studies on these deficits have yielded more consistent results. In the motor cortex, short interval intracortical inhibition (SICI) has been repeatedly found to be reduced in children with ADHD in a passive (resting) condition [17], [18], [19], [20]. It has been noticed that a reduced SICI is not specific for ADHD but can also be found in other neurological and psychiatric disorders (e.g., Parkinson’s disease, obsessive-compulsive disorder [16]).

SICI and other components of cortical excitability can be measured noninvasively by transcranial magnetic stimulation (TMS). By applying single-pulse TMS to the motor cortex, motor evoked potentials (MEP) can be measured in contralateral extremity muscles (e.g., the m. abductor digiti minimi) [21], [22], [23], [24]. Using paired-pulse TMS, two pulses are delivered through the same coil. This paradigm allows for the study of inhibitory and facilitatory effects within the motor cortex [25]. The intensity and the interstimulus interval (ISI) between subthreshold conditioning and the supra-threshold test stimulus influence the outcome. This relationship exists because different circuits are recruited, and the time constants for the activated circuits might differ [22]. An ISI between 1 to 4 ms generates a diminished MEP compared to single-pulse MEP amplitude. This inhibition of the MEP amplitude is called short intracortical inhibition (SICI). Intracortical facilitation is the increase in amplitude that occurs with an ISI of 6 to 25 ms [24].

These phenomena are ostensibly mediated in cortical regions, as intracortical inhibition and facilitation are seemingly the result of the activation of separate neuronal circuits [26]. Based on the effect of different medications, SICI is thought to reflect the activity of GABA_A_ergic inhibitory interneurons within the motor cortex [27]. It is also modulated by dopamine [28], e.g., methylphenidate alters SICI in a dose- and gene-dependent manner and could exert its effects directly in the motor cortex by dopaminergic inputs [20].

TMS is not only a useful tool for studying motor system excitability during rest, but also when individuals are actively engaged in a cognitive or motor task [24], [29]. In a laboratory setting, inhibitory processes in an active motoric condition can be examined with the go/nogo task. For each trial, either a go or a nogo stimulus is presented. Subjects have to respond to the go stimuli or inhibit a prepared motoric act in nogo trials, respectively. Several studies with adults have examined motor cortex excitability in an active task condition. A reduction of inhibition was measured in go trials immediately prior to electromyography (EMG) onset [30], [31]. In nogo trials, an increase in SICI [32], [33], [34] and a decrease in single-pulse MEP amplitudes [35], [36], [37] were found at a time corresponding to the mean reaction time in go trials.

The main aim of this study was to determine SICI in an active response inhibition task in children with ADHD compared to typically developing (TD) controls. To the best of our knowledge, this study is the first evaluation of motor excitability at different latencies before, during and after the voluntary suppression of movement in this age cohort. We predict that distinct patterns of excitability would be observed between children with ADHD and TD controls. Because SICI at rest depends on the level of hyperactivity and impulsivity [18], [38], we also predicted that different patterns would exist for children with lower and higher symptomatic occurrence.

## Methods

### Subjects

43 children with ADHD (35 males) and 29 typically developing control subjects (24 males) aged 9–14 years attended the study. Both groups did not differ with respect to age, IQ and the distribution of hand preference (assessed via the Edinburgh Handedness Inventory [39]).

After performing a median split, the ADHD group was subdivided into two groups (H/I-low ≤16.5; H/I-high >16.5) according to individual levels of hyperactivity and impulsivity, as assessed by the German ADHD rating scale, FBB-HKS [40]. Accordingly the groups differed on the H/I subscale but not on the inattention scale. In Table 1, the main sample characteristics are summarised for the three groups (TD, H/I-low and H/I-high). Only those children are included who had TMS data of sufficient quality. All patients fulfilled the DSM-IV criteria for ADHD Combined type [41].

**Table 1:** Demographical and clinical data for the control, H/I-low and H/I-high group and statistical comparison (***p<.001).

	Controls	H/I-low		H/I-high	ANOVA		
	(C)	(L)		(H)	F (2, 51)		Post Hoc Tests
**N**	**24**	**15**	**14**				
**Demographical data**							
Age, years	**11.99** (1.54)	**12.56** (1.33)	**11.72** (1.33)		1.4	–	
Boys	**75%**	**80%**	**93%**				
IQ	**110** (14)	**106** (14)	**110** (14)		0.5	–	
**ADHD rating scale (FBB-HKS)**							
Total Score	**5.33** (3.33)	**25.87** (6.27)	**40.04** (7.16)		191.7***	C<L^***^, C<H^***^, L<H^***^	
Subscale Inattention	**3.46** (2.69)	**15.43** (5.17)	**17.75** (5.32)		63.0***	C<L^***^, C<H^***^	
Subscale Hyperactivity/Impulsivity	**1.88** (1.94)	**10.63** (3.19)	**22.29** (3.58)		234.1***	C<L^***^, C<H^***^, L<H^***^	

Subjects were recruited by local professionals and via the outpatient clinic of the Department of Child and Adolescent Mental Health in Erlangen. A medical assistant or a clinical psychologist assigned diagnoses based on clinical interviews with parents and patients, which were supervised by a board-certified child and adolescent psychiatrist. The FBB-HKS was used to review ADHD symptoms. All individuals of the ADHD group fit the criteria for the combined subtype and displayed at least 0.5 point scores for the hyperactivity and impulsivity subscales and a total score of at least 1 point.

Subjects with comorbid dyslexia or oppositional defiant disorder were allowed to participate. Children with other comorbid diagnoses, particularly those affecting motor system excitability (e.g., tic disorder [42]) and neural processing in response control tasks (e.g., conduct disorder [43]) were excluded. Neurological impairments and learning disability (IQ <80), which was assessed with the Hamburg-Wechsler Intelligence Test for Children (third edition), were considered as exclusionary criteria.

Due to the short half-life of methylphenidate (2–4 hours), children taking this stimulant medication could participate (N = 11) after a washout period of at least 48 hours. Other medications were not allowed.

Individuals with exclusion criteria for TMS, such as epilepsy, brain injury or any brain disturbance (vascular, inflammatory or degenerative) were not included. Electroencephalogram (EEG), electrocardiogram examinations and a medical interview were performed to exclude participants with cardiovascular diseases, low seizure threshold or predisposition for syncope [44], [45].

The subjects of the control group were recruited from different non-clinical settings (e.g., schools and sports clubs). The children and their parents were subjected to identical screening procedures as the children with ADHD, i.e., a medical assistant or a clinical psychologist conducted clinical interviews with parents and patients, which were supervised by a board-certified child and adolescent psychiatrist. Control subjects were included in the study if they had a FBB-HKS total score lower than 0.5 and did not possess any neurological or psychiatric disorders.

Written informed parental consent and verbal participant assent were obtained from all subjects. The experiment was performed in accordance with the Declaration of Helsinki and was approved by the Ethics Committee of the University of Erlangen-Nürnberg.

### Procedure

Single- and paired-pulse TMS was given with a hand-held figure eight (diameter of one wing = 70 mm) connected to a Magstim® BiStim unit with two Magstim® 200^2^ stimulators (Magstim, Whitland, UK).

The optimal site for stimulation over the left motor cortex was determined for eliciting MEPs in the m. abductor digiti minimi of the right hand. This site was marked with a felt pen. The resting motor threshold (RMT) was defined as the minimum stimulus intensity that produced a liminal motor evoked response (approximately 50 µV in 50% of 10 trials) in the relaxed target muscle. The intensity of the conditioning stimulus was set to 75% of RMT, and the supra-theshold stimulus was adapted (with a maximum of 20% above RMT) to evoke a MEP with a mean peak-to-peak amplitude of 1 mV when given alone.

### Go/nogo Task and TMS Conditions

**Figure 1:**
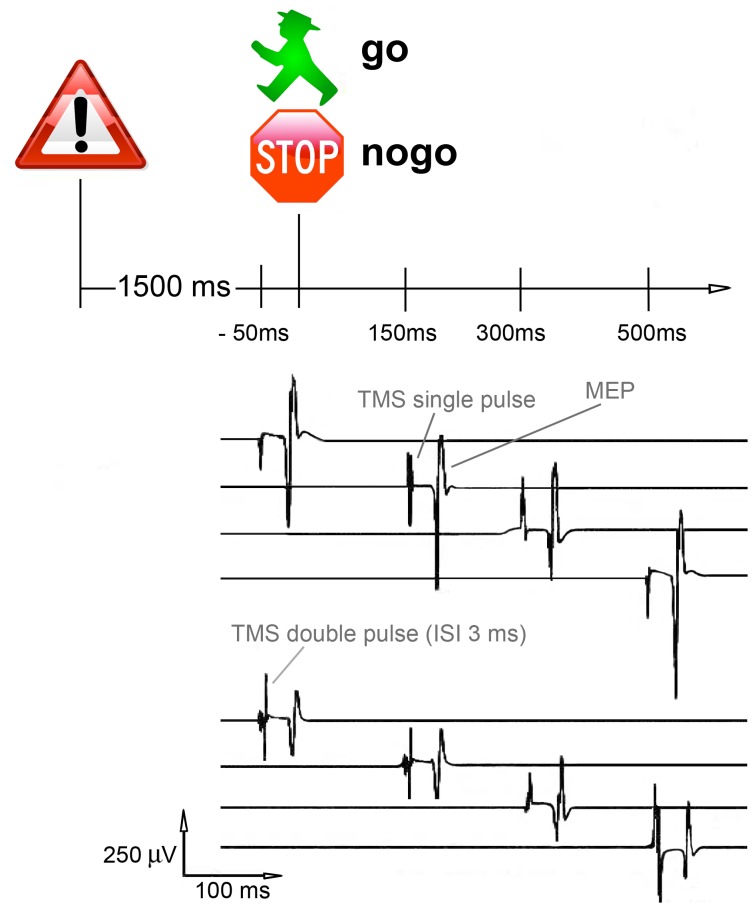
Illustration of the go/nogo task (S1–S2 paradigm). TMS stimuli (single-pulses or double-pulses with an interstimulus interval of 3 ms) were presented 50 ms before or 150, 300 or 500 ms after the onset of the S2 stimulus.

The go/nogo task was implemented using Presentation® (Version 11.0; Neurobehavioral Systems, Albany, CA, USA). The task consisted of four experimental blocks with 48 trials per block. Each trial started with the presentation of a warning stimulus (a danger traffic sign; S1, 250 ms duration), which was followed by a test stimulus (S2, 250 ms duration). S2 was either a red stop sign (nogo condition) or the green figure commonly found in pedestrian traffic lights that signifies safe passage (go condition). The ratio of go to nogo trials was set to 1∶1. The interval between S1 and S2 was 1500 ms, the intertrial interval (S1–S1) was 5000±1000 ms (see Fig. 1).

To increase participant motivation and influence go stimuli reactions to the greatest degree, a financial reward (6 cents per trial) was given for correct responses in a certain time window after the go stimulus. This time window was based on a tracking algorithm and dynamically adjusted to the 75^th^ percentile of reaction times (go trials) of every previous block and the practice block, respectively.

In the case of a wrong reaction (no reaction to a go stimulus within 1500 ms or reaction to a nogo trial), the same amount of money was subtracted.

The participants received acoustic feedback for correct (the sound of a cash register opening) and incorrect (honk sound) responses.

Presentation® was used to trigger the magnetic stimulators. Either a single-pulse or a double-pulse (inter-stimulus-interval 3 ms) was delivered at various latencies after S2 (150 ms, 300 ms or 500 ms) or 50 ms before S2. Catch trials were interspersed in the intertrial period, which served as a control condition.

There were 16 different pulse x latency x go vs. nogo conditions (e.g., single-pulse x latency of 300 ms x nogo trial) and 2 conditions (single- and double-pulse) for catch trials. Each condition appeared three times and varied randomly within each block (48 trials and 6 catch trials per block).

### Experimental Design

**Figure 2:**
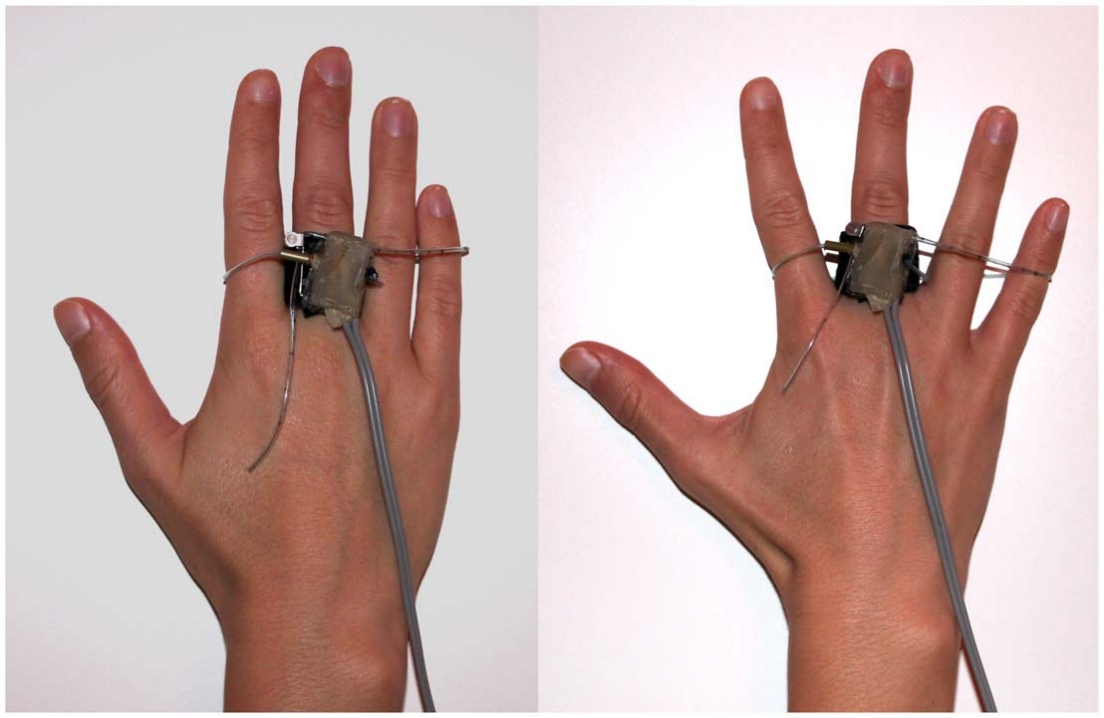
Switch to register reactions. Spreading of the hand with the m. abductor digiti minimi involved. Left side: switch off. Right side: switch on.

Children were invited to sit on a comfortable, straight-backed chair with armrests to minimise artefactual movement and asked to relax as much as possible during the whole experiment. A 17 inch monitor was placed 90 cm in front of them at eye level, and subjects were instructed to react to go stimuli by spreading the fingers of the right hand. A switch was attached to the index finger for use as an input device that transmitted the responses to a connected PC. The middle, ring and little finger were linked via a plastic loop to the switch, which was triggered when the fingers were spread (see Fig. 2). The task was introduced to the participants, and they were acclimated to the task with two short practice blocks, one with TMS and the other without. These practice sessions helped prepare subjects for TMS stimulation, which could interfere with the execution of the go/nogo test.

For each participant, one out of four experimental blocks was randomly applied without TMS.

### Data Recording and Pre-processing

A BrainAmp® recording system (standard BrainAmp amplifier; Brain Products, Gilching, Germany) was used for data acquisition. EMG was recorded from the abductor digiti minimi muscle of the right hand at a sampling rate of 5000 Hz. Filter bandwidth was set to 8–1000 Hz.

EEG activity was recorded simultaneously, but will not be reported here. A standard EEG cap (easycap, Inning a. Ammersee, Germany) was worn throughout the experiment but without electrodes at the site of TMS stimulation (particularly without C3).

### Data Processing and Analysis

The recorded EMG data were subdivided into segments with lengths of 300 ms (from 150 ms before until 150 ms after the TMS pulse) with one segment per trial. Only trials with a correct behavioural response were considered for further analyses.

If intracortical excitability is measured at rest, the evoked MEP amplitude has to typically fulfil a peak-to-peak amplitude criterion between 500–1500 µV, or the trial is removed (e.g., [17]). For the TMS data analysis for the response inhibition task, we adopted the procedure used by Kratz et al. [33] and ran two peak detections. First, we excluded all segments with an initial EMG activity larger than 50 µV in a time window of 110 to 25 ms before the TMS pulse to avoid trials with pre-tension of the right hand muscles.

A second peak detection was processed to calculate the peak-to-peak MEP amplitude of every segment in a time range of 20 to 50 ms after magnetic stimulation. Only trials with amplitude between 70 µV and 4000 µV were included, which eliminated outlier values that were due to artefacts, background EMG or technical problems.

We calculated relative MEP amplitudes (mean amplitude for each pulse x latency x go/nogo task condition) for a better comparison of the groups. Therefore, the arithmetic mean of the single-pulse MEPs of the control condition (catch trials) was defined as 100 percent.

The registered mean reaction time for go trials was ∼380 ms (see Table 2) but the EMG activity started already ∼100 ms before the response was triggered. This means that EMG activity was present in go trials from ∼280 ms after S2 and therefore in most of the go trials where TMS was applied at latencies of 300 ms or 500 ms. For this reason, go trials with these TMS latencies were not analysed further.

**Table 2:** Behavioural performance of the go/nogo task, subdivided in blocks with TMS and the block without TMS, and statistical comparison between the three groups.

	Controls	H/I-low	H/I-high	ANOVA	
	(C)	(L)	(H)	F (2, 51)	Post Hoc Tests
**Blocks with TMS** **Performance level (go/nogo-task)**					
Reaction times, ms	**384** (59)	**383** (59)	**388** (39)	0.04	–
Reaction time variability, ms	**74** (23)	**81** (25)	**83** (21)	0.7	–
Commission errors	**0.88** (0.75)	**0.64** (0.75)	**1.14** (1.65)	0.8	–
**Block without TMS** **Performance level (go/nogo-task)**					
Reaction times, ms	**375** (62)	**369** (61)	**381** (33)	0.2	–
Reaction time variability, ms	**71** (37)	**79** (37)	**81** (33)	0.4	–
Commission errors	**0.48** (0.67)	**0.27** (0.46)	**0.57** (1.34)	0.5	–

### Statistical Analysis

Performance data (commission errors = responding during nogo trials, mean reaction time, reaction time variability) for blocks with and without TMS were analysed separately by univariate analysis of variance (ANOVA) with group (TD vs. H/I-low vs. H/I-high) as between-subject factor. Paired t-tests were used to evaluate differences in task performance between blocks with and without TMS.

We performed univariate ANOVA to determine differences in RMT, MEP amplitude of the passive control condition, stimulus intensity and SICI at all latencies between the TD, H/I-low and H/I-high groups (between-subject factor).

Pearson’s correlation coefficients were calculated to assess the strength of the linear relationship between SICI and the levels of hyperactivity and impulsivity.

The SICI change (ratio of conditioned and unconditioned MEP response) and relative MEP amplitudes over different latencies for the three groups (between-subject factor) were analysed by using repeated measures ANOVA, as well as differences in go and nogo trials (within-subject factor) for the three groups.

We utilised one-way ANOVA to analyse time course dynamics over the different latencies (within-subject factor) of motor inhibition and corticospinal excitability for each group.

Paired t-tests for within-subject analysis were conducted, and Bonferroni’s post hoc tests were used to assess between-subject differences when interactions were significant.

Go and nogo trials were analysed independently, and the main focus was on nogo trials. Data are presented as means ± SD, and a p value of <0.05 (<0.0167 in Bonferroni’s post hoc analysis) was considered to indicate significance.

The degrees of freedom were adjusted with the Greenhouse-Geisser correction when appropriate. IBM SPSS Statistics 19.0 was used for statistical analyses.

## Results

The recruited sample included 72 children (29 TD, 22 H/I-low and 21 H/I-high).

The procedure was well tolerated by 68 participants. Three individuals (1 TD and 2 H/I-high) felt uncomfortable with the TMS stimulation and dropped out of the study. Physiology data could not be obtained in 9 subjects (2 TD, 3 H/I-low and 4 H/I-high), due to high thresholds. After artefact inspection, data from 53 children (24 TD, 15 H/I-low and 14 H/I-high) remained in the study sample.

### Performance Data

The behavioural performance of the go/nogo task was subdivided into blocks with TMS and the block without TMS (see Table 2).

The three groups did not differ significantly in commission errors, reaction time and reaction time variability in any of the four blocks. Significant effects only appeared between the blocks with and without TMS. All three groups made significantly fewer commission errors in the block without TMS (TD group: t(22) = −2.48; p<.05; H/I-low: t(14) = −2.24; p<.05; H/I-high: t(13) = −2.75; p<.05). The TD (t(22) = −2.11; p<.05) and H/I-low (t(14) = −2.5; p<.05) groups reacted slightly, but significantly, faster in the block without magnetic stimulation. No differences were found for the reaction time variability.

### TMS Parameters

No significant neurophysiological differences were obtained between the three groups for RMT (TD: 51.5±8.6%, H/I-low: 49.1±9.6%, H/I-high: 52.8±7.0%; F(2, 50) = .7, p = .49), MEP amplitude of the passive control condition (TD: 1.2±.5 mV, H/I-low: 1.3±.6 mV, H/I-high: 1.0±.5 mV; F(2, 50) = 1.3, p = .3) or stimulus intensity (TD: 69.6±11.1%, H/I-low: 65.4±13.9%, H/I-high: 71.4±8.4%; F(2, 50) = 1.1, p = .34). There was no correlation between threshold and ADHD symptom rating scores.

### TMS Data – Double-pulse

**Figure 3:**
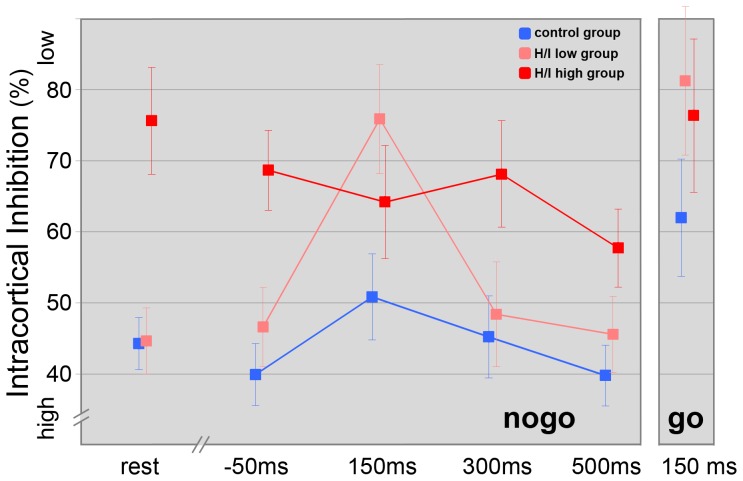
Intracortical inhibition over the course of the nogo trials, for the go-150 ms condition and the passive control condition. For each of the three groups, mean ± SE is depicted.

Univariate ANOVAs revealed significant differences for SICI between the three groups at all latencies (rest, SICI −50, nogo SICI 150/300/500) (see Table 3/Fig. 3).

**Table 3:** Resting motor threshold and SICI for the passive control condition and the different latencies over the course of the nogo trials (higher SICI scores indicate less inhibition) and statistical comparison between the three groups (*p<.05, **p<.01,***p<.001).

	Controls	H/I-low	H/I-high	ANOVA	
	(C)	(L)	(H)	F (2, 51)	Post Hoc Tests
**RMT, % of max. stimulator output**	**51.5** (8.6)	**49.1** (9.6)	**52.8** (7.0)	0.7	**–**
**SICI control, %**	**44** (16)	**44** (14)	**76** (24)	16.3***	H<C***, H<L***
**SICI –50 ms, %**	**40** (17)	**47** (14)	**69** (32)	8.4**	H<C***, H<L*
**SICI 150 ms, %**	**51** (25)	**76** (35)	**64** (31)	3.4*	L<C*
**SICI 300 ms, %**	**45** (27)	**48** (30)	**68** (28)	3.2*	H<C*
**SICI 500 ms, %**	**40** (19)	**46** (19)	**58** (24)	3.5*	H<C*

**Figure 4:**
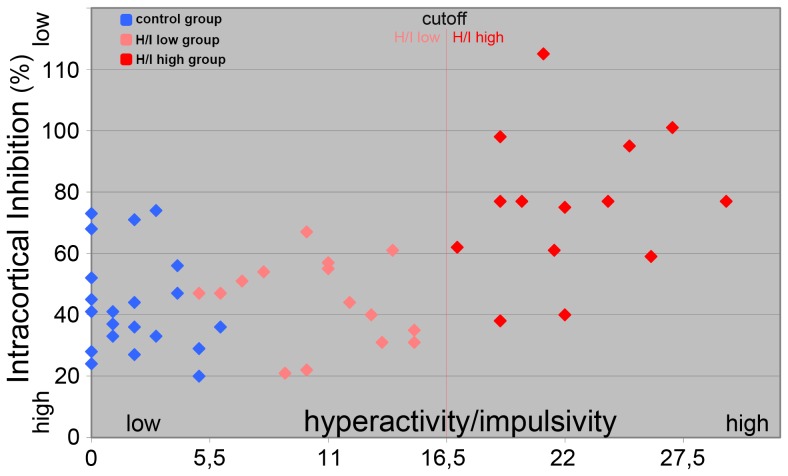
Scatter plot illustrating the correlation between intracortical inhibition at rest and the level of hyperactivity/impulsivity (ADHD rating scale, FBB-HKS).

The SICI in the resting condition (SICI control) and 50 ms before S2 (SICI –50) was significantly reduced in the H/I-high group relative to the TD- and H/I-low group (see Table 3). However, there was no difference between the TD group and the H/I-low group at this time point (t(37) = −1.27; p = .21) and in rest (t(37) = −.02; p = 1.0). Therefore we found significant correlations over all three groups (r = .54; p<.001) and within the ADHD group (r = .58; p = .001) between a higher SICI in rest and less occurrence of hyperactivity and impulsivity (see Fig. 4).

300 and 500 ms after S2 (SICI 300/SICI 500) SICI was significantly reduced in the H/I-high relative to the TD group and significantly reduced in the H/I-low group compared to TD children at a latency of 150 ms (SICI 150).

Repeated measures ANOVA that compared the three groups’ nogo-patterns of excitability yielded significant main effects for the factors “latency” (F(2, 50) = 4.6, p = .004), “group” (F(2, 50) = 8.8, p = .001) and for the interaction between these two factors (F(2, 50) = 2.7, p = .015). Bonferroni post hoc testing indicated significant differences between the TD and H/I-high groups (p<.001) and a trend for the H/I-low and H/I-high groups (p = .053) (see Fig. 3).

Group-specific analysis of nogo-patterns revealed a significant increase in SICI from control to 500 ms after S2 in the H/I-high group and a trend for a linear latency effect (T-lin(1, 13) = 3.9, p = .07).

In the H/I-low group, SICI decreased significantly at a latency of 150 ms and increased significantly from that time point to 300 and 500 ms. The ANOVA obtained a significant quadratic latency effect (T-quad(1, 14) = 14.4, p = .002) for this group.

A significant decrease in SICI at a latency of 150 ms followed by an increase to 500 ms and a trend for a quadratic latency effect (T-quad(1, 23) = 3.2, p = .09) could be found in the TD group.

### TMS Data – Single-pulse

Repeated measures ANOVA that compared the three groups’ patterns of corticospinal excitability revealed a significant main effect for the factor “latency” (F(2, 49) = 3.1, p = .04), but no “group” (F(2, 49) = .9, p = .4) or interaction effects between these two factors (F(2, 49) = 1.2, p = .33) were observed.

**Figure 5:**
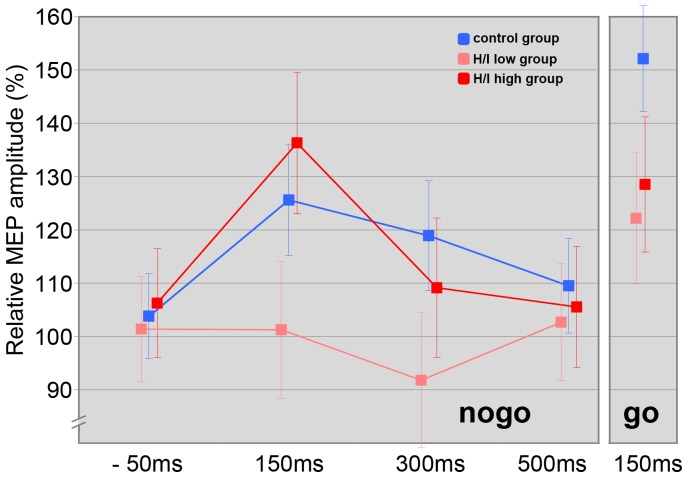
Overall excitability over the course of the nogo trials, for the go-150 ms condition and the passive control condition. For each of the three groups, mean ± SE is depicted.

Group-specific analysis of relative single-pulse MEP amplitudes over the time course of the task obtained a significant quadratic latency effect (T-quad(1, 22) = 5.5, p = .03) for the TD group (see Fig. 5). A significant cubic latency effect (T-cubic(1, 13) = 5.0, p = .04) and a trend for a quadratic latency effect (T-quad(1, 13) = 3.8, p = .07) was obtained for the H/I-high group (see Fig. 5).

### TMS Data – go vs. nogo Trials at a Latency of 150 ms

Repeated measures ANOVA was used to analyse differences in go and nogo trials (within-subject factor) at a latency of 150 ms for the three groups.

In double-pulse TMS a significant “group” effect (F(2, 50) = 3.6, p = .03) but no effect for the factor “go/nogo” (F(2, 50) = 2.0, p = .16) or an interaction effect (F(2, 50) = .1, p = .92) could be obtained (see Fig. 3).

Single-pulse TMS results revealed a significant main effect for the factor “go/nogo” (F(2, 50) = 4.3, p = .04), no effect for the factor “group” (F(2, 50) = 1.3, p = .29) and a trend towards significance for the interaction between these factors (F(2, 50) = 2.7, p = .08).

Concerning single-pulse MEPs, Bonferroni post hoc paired t-tests yielded significant differences between go and nogo trials at a latency of 150 ms for the TD (t(23) = 2.7; p<.01) and H/I-low group (t(14) = 2.2; p<.05) (see Fig. 5).

### TMS Data – Change of SICI from −50 to 150 ms in go and nogo Trials

Group-specific analysis revealed a significant decrease in SICI from −50 ms to 150 ms in nogo trials for the TD (t(23) = −2.3; p<.05) and the H/I-low group (t(14) = −3.6; p<.01). In go trials, a significant decrease in SICI could be obtained from −50 ms to 150 ms for the TD (t(23) = −2.91; p<.01) and H/I-low group (t(14) = −2.78; p<.05) (see Fig. 3).

## Discussion

In the present study, we investigated differences in motor excitability (particularly short interval intracortical inhibition) in typically developing control children and two groups of ADHD children with either a lower or a higher level of hyperactivity and impulsivity. In contrast to previous studies that analysed motor excitability in a resting condition, TMS was applied during a go/nogo task at stages of response preparation, activation and suppression, respectively. We compared the SICI measured during these stages to the SICI determined in a resting condition, as applied between the task trials.

Using motivational incentives, performance (mean reaction time, reaction time variability and commission errors) of the go/nogo task did not differ between the three groups. As hypothesised, time course analysis revealed different patterns of excitability for the three groups, and our methodical approach helped to identify specific inhibitory patterns for children with different levels of hyperactive and impulsive symptoms.

### Factors Affecting Task Performance

#### Influence of TMS stimulation

All three groups showed slight performance impairments in TMS rounds compared to the block without TMS, which has also been reported in previous studies [28], [36]. The influence of TMS on task performance could result from a combination of effects caused by magnetic stimulation and acoustic and visual distraction [46]. Motoric constraints imposed by a simultaneous MEP elicited at the hand might have limited the subjects’ ability to react as quickly as possible to the go stimuli. In a time frame of 40-ms after magnetic stimulation, voluntary EMG activation can’t be observed, which results in delayed reaction time [36]. Alternatively, the presence of a clicking sound from the coil could have distracted the subjects’ attention. Because the differences in task performance (TMS vs. non-TMS) are comparable in all groups, this putative distracting effect did not differentially influence the groups.

#### Motivational aspects

We aimed for the three groups to achieve similar neuropsychological task performance such that differences obtained at the neuronal level did not simply reflect bad task performance or were not due to poor task engagement.

According to the dual-deficit hypothesis [47], motivational deficits can function in certain subgroups of patients that are under-aroused and consequently not performing at optimum levels [48]. Although some studies have found contingencies to have a comparable positive effect on motivation and, consequently, on task performance in ADHD and typically developing control children [7], [48] or to have no significant effects on inhibition deficits [49], most studies have shown that the effect related to task output is more dominant in ADHD, especially when highly intensive incentives were used [50].

Thus, we attempted to boost performance in all subjects to the highest possible level by offering monetary incentives for correct responses. Because all groups showed a comparable task performance (even concerning reaction time variability), it can be proposed that the obtained TMS results were not due to group differences in motivation. Otherwise the lack of a no-incentive condition can be seen as a limitation in our study.

### General Findings in Motor Excitability

#### Overall excitability

In TD children, statistics revealed a quadratic latency effect for corticospinal excitability over the course of the *nogo trials*: single-pulse MEP amplitudes (see Fig. 5) increased significantly from −50 ms to a latency of 150 ms after S2 but decreased to the levels observed prior stimulus presentation at ∼500 ms. In healthy young adults, similar patterns of overall excitability were found in multiple studies [35], [36]. However, corticospinal excitability in healthy adolescents had already decreased beyond baseline levels between 100 and 200 ms after the presentation of a nogo stimulus, which was interpreted as a result of the strong inhibition of the corticospinal pathway after the decision to inhibit the prepared response. Therefore, on a cortical level, the decision to inhibit a motor response is made earlier in adults, which is due to developmental delays in children that were also reflected in higher reaction times. Additionally, the modulation of corticospinal excitability to changing task demands might be delayed in children. Overall excitability returned to base level at ∼500 ms, whereas the decision to inhibit a prepared response was made less than 150 ms after the nogo stimulus. In this respect, we consider this time interval (0–150 ms) to be the response activation phase, which merges into the suppression or execution phase. This second phase potentially started before 150 ms, as evidenced by significantly enhanced MEP single-pulse MEP amplitudes in go trials compared to nogo trials at latencies of 150 ms, which reflected an initial adaptation of corticospinal excitability.

Increases of single-pulse MEP amplitudes during *response activation* were reported in several studies in young healthy adults, starting from ∼120 ms before EMG onset [30], [31] and 100–300 ms after the go stimulus [35], [36], respectively. The effect is thought to depend on cortical and spinal mechanisms and probably reflect increased activation of pyramidal neurons within the motor cortex, which decreases the excitability threshold of the pyramidal tract to facilitate the motor command [51].

Less than 150 ms was required for the inhibition process to counteract the go signal activation during successful response inhibition, which manifested as a decrease of MEP amplitudes. It may be concluded that, similar to the horse race model in stop tasks [52], processes of primary response activation and secondary inhibition were independently activated and overlapped in nogo trials. Depending on which process prevails, the prepared response was either executed or inhibited.

#### Short intracortical inhibition

In go and nogo trials, response activation was accompanied by a significant decrease of intracortical inhibition in the period from −50 ms towards a latency of 150 ms in TD children (see Fig. 3). Afterwards, SICI increased in nogo trials (Please notice that a higher SICI score indicates less inhibition and vice versa). Previous studies described this increase towards a time point that corresponded to the mean reaction time in go trials in healthy adults [33], [34].

Considering all groups, there was a tendency for increased SICI at a latency of 150 ms in nogo trials compared to go trials, which also implied that the process of voluntary suppression of a prepared response had already begun at this point. This result is in accordance with previous TMS data in adults that demonstrated higher intracortical inhibition in nogo trials compared to go trials at a latency of 120 ms [33] and at the time of EMG onset [30], [34], respectively.

SICI is thought to reflect the activity of inhibitory interneurons in the motor cortex, which contribute to the volitional suppression of a prepared response in nogo trials [37]. Thus, these interneurons become increasingly activated after the decision to withhold the response.

### Motor Excitability in ADHD

#### ADHD subgroups

Though we only included children with ADHD of the combined type (DSM-IV criteria), we had decided not to build one ADHD group but two subgroups according to the level of hyperactivity/impulsivity. This subdivision was mainly based on the finding of previous TMS studies that found less SICI to be correlated with higher severity of hyperactivity [18], [19], [38]. This correlation could also be confirmed for our sample of children with ADHD considering SICI in the passive control condition. As a consequence, children of the H/I-low group showed the same level of SICI than TDs, but differed from the H/I-high group, for which SICI was significantly reduced.

Hence, it seems favourable for studying brain phenotypes to take the different SICI levels at rest into account instead of averaging over the complete ADHD group [10].

Particularly concerning SICI, qualitatively different patterns were obtained for these ADHD subgroups. For overall excitability, only slighter differences were obtained.

#### ADHD group with a higher level of hyperactivity and impulsivity

In contrast to TD children, for whom a quadratic pattern was revealed, H/I highs showed a linear increase of SICI over the course of the nogo trials (see Fig. 3). As mentioned above, children from the H/I-high group showed a diminished SICI in the passive control condition. SICI was about the same in the movement preparation phase, 50 ms before the go or nogo-stimulus as in the control condition.

Whereas SICI decreased in the TD and the H/I-low group towards a latency of 150 ms in go trials compared to premovement phase at a latency of −50 ms, SICI in the H/I-high group remained on the same low level at 150 ms.

Hence, SICI in children of the H/I high-group appeared similar at rest relative to movement activation phase (immediately before a movement), which represents a disinhibited state. A ceiling effect cannot account for this phenomenon since as e.g. shown in Kujirai et al. [25] where the effects of different conditioning stimulus intensities were considered, reduced inhibition can even turn into facilitation. So, our finding in the H/I high group might help to elucidate the hypermotoric behaviour of these children.

After the decision to restrain the response had been made, intracortical inhibition increased significantly, which reflected stronger activation of inhibitory interneurons within the motor cortex.

These findings suggest that children with a higher level of hyperactivity and impulsivity are able to engage compensatory neural mechanisms to allocate more inhibitory resources for an adequate task performance.

However, SICI was still reduced at the latency of 500 ms compared to TD children (see Fig. 3). Thus, children with higher levels of hyperactivity and impulsivity had inhibitory deficits (decreased activation of inhibitory interneurons in the motor cortex) that could be related to a dysregulation within the mesocorticolimbic dopamine system [38].

#### ADHD group with a lower level of hyperactivity and impulsivity

Children of the H/I-low group displayed a similar quadratic pattern of intracortical inhibition over the course of the nogo trial as children in the TD group (see Fig. 3). However, disinhibition was more pronounced at a latency of 150 ms (in go and nogo trials) though they were able to modulate SICI back to a level similar to TD children, demonstrating a comparable task performance. In the response activation phase, especially when reaction swiftness is important for monetary incentives, inhibitory control was abandoned in favour of the fastest possible reaction. Thus, reduced motor control in children of the H/I-low group in these specific situations (as reflected by significantly diminished SICI at a latency of 150 ms compared to TD children) might be characteristic for the H/I-low group.

Concerning overall excitability, a different activation pattern was observed for H/I-low children in nogo trials. Whereas TD children showed a quadratic pattern with an increase of unconditioned MEP amplitudes at a latency of 150 ms that was followed by a decrease towards 500 ms, corticospinal excitability did not change in the H/I-low group (see Fig. 5). Thus, this finding also suggests a differential organisation of the suppression of a movement in the the H/I-low group at the neuronal level.

#### Limitation

A limitation of our study might be the decision to use a 3-ms interstimulus interval between conditioning and test stimulus as well as a conditioning stimulus intensity of 75%. For example, the data might be biased toward an influence of intracortical facilitation [53]. In addition, it could have been better to measure SICI at constant response, using a threshold tracking procedure [54]. However, the finding of a reduced SICI in children with ADHD had been reported in several studies using different intensities for the conditioning stimulus and different interstimulus intervals [17], [19], [20], [38].

Recent methodological studies addressing SICI (e.g., Vucic [55]) also used a 3-ms interstimulus interval and comparable stimulus intensities depending on resting motor threshold. So, in our opinion, the settings used in our study should yield valid results concerning intracortical inhibition.

### Conclusions

Time course analysis in a response inhibition task identified different patterns of motor excitability for children with ADHD and a higher or lower level of hyperactivity and impulsivity compared to typically developing children.

In the H/I-low group, response activation was accompanied by a marked decrease of SICI, which might reflect a reduced motor control due to the subsequent fast motor response. For the children of the H/I-high group, findings indicate an inhibitory deficit in the motor cortex. These children seem to be in a less controlled state at rest due to a reduced activation of inhibitory interneurons. These inhibitory interneurons show about the same activation at rest as during response activation, which might be related to the hypermotoric behaviour of those children with ADHD.

Therefore, our methodological approach may be considered as a significant contribution to a better understanding of dysfunctional motor control and behaviour in children with ADHD which might help identify causal pathways to this disorder. The differences in H/I highs and H/I lows provide further evidence for ADHD subtypes concerning motor control.

In future studies, it will be interesting to analyse possible associations of SICI alterations and specific genetic variations in the dopamine transporter (DAT1) that is among others possibly related to dysregulation within the mesocorticolimbic dopamine system [56]. Moreover, future studies on the underlying processes of motor response inhibition might provide further insight by combining TMS and event-related potentials (ERPs). In a recent study with healthy adults [57], inhibition-related TMS measures (e.g., SICI) and the contingent negative variation explained about 85% of the variance of the nogo P3 in a go/nogo task paradigm. These results illustrated an interplay between different neurophysiological processes that act in concert to successfully inhibit a prepared movement. These ERP and TMS parameters have repeatedly been reported to be altered in children with ADHD, which suggests that this venue could be of particular value for studying ADHD.
